# Scaling up malaria intervention “packages” in Senegal: using cost effectiveness data for improving allocative efficiency and programmatic decision-making

**DOI:** 10.1186/s12936-018-2305-6

**Published:** 2018-04-10

**Authors:** Sophie Faye, Altea Cico, Alioune Badara Gueye, Elaine Baruwa, Benjamin Johns, Médoune Ndiop, Martin Alilio

**Affiliations:** 1 Health Finance and Governance Project, Abt Associates, 6130 Executive Boulevard, Rockville, MD 20852 USA; 2 Senegal National Malaria Control Programme, Dakar, Senegal; 3 United States Agency for International Development, President’s Malaria Initiative, Washington, D.C., USA

**Keywords:** Malaria, Elimination, Cost effectiveness, Programme scale up, Senegal

## Abstract

**Background:**

Senegal’s National Malaria Control Programme (NMCP) implements control interventions in the form of targeted packages: (1) scale-up for impact (SUFI), which includes bed nets, intermittent preventive treatment in pregnancy, rapid diagnostic tests, and artemisinin combination therapy; (2) SUFI + reactive case investigation (focal test and treat); (3) SUFI + indoor residual spraying (IRS); (4) SUFI + seasonal malaria chemoprophylaxis (SMC); and, (5) SUFI + SMC + IRS. This study estimates the cost effectiveness of each of these packages to provide the NMCP with data for improving allocative efficiency and programmatic decision-making.

**Methods:**

This study is a retrospective analysis for the period 2013–2014 covering all 76 Senegal districts. The yearly implementation cost for each intervention was estimated and the information was aggregated into a package cost for all covered districts. The change in the burden of malaria associated with each package was estimated using the number of disability adjusted life-years (DALYs) averted. The cost effectiveness (cost per DALY averted) was then calculated for each package.

**Results:**

The cost per DALY averted ranged from $76 to $1591 across packages. Using World Health Organization standards, 4 of the 5 packages were “very cost effective” (less than Senegal’s GDP per capita). Relative to the 2 other packages implemented in malaria control districts, the SUFI + SMC package was the most cost-effective package at $76 per DALY averted. SMC seems to make IRS more cost effective: $582 per DALY averted for SUFI + IRS compared with $272 for the SUFI + IRS + SMC package. The SUFI + focal test and treat, implemented in malaria elimination districts, had a cost per DALY averted of $1591 and was only “cost-effective” (less than three times Senegal’s per capita GDP).

**Conclusion:**

Senegal’s choice of deploying malaria interventions by packages seems to be effectively targeting high burden areas with a wide range of interventions. However, not all districts showed the same level of performance, indicating that efficiency gains are still possible.

## Background

In 2015, there were approximately 212 million cases and 429,000 deaths from malaria worldwide [1], and 90% of the cases occurred in sub-Saharan Africa [1]. As countries strive to achieve malaria elimination with limited resources, it is critical to understand the cost-effectiveness of interventions that they are using or could use to prevent or treat malaria. However, policymakers and planners lack access to reliable and context-specific cost and cost-effectiveness analysis (CEA) results to identify efficient combinations of interventions leading to rapid progress toward malaria elimination.

A systematic review of the cost and cost-effectiveness of malaria control interventions conducted by White et al. found that most existing evidence is trial-based, rather than reflecting a real implementation context. CEAs either compared single interventions with a before-intervention situation or compared several stand-alone interventions [2]. A more recent review by Gunda and Chimbari concluded that there was still a lack of information on the cost effectiveness of combined malaria interventions in most low- and medium-income countries where the burden of malaria is highest [3]. Both White et al. and Gunda and Chimbari found few studies examining cost-effectiveness of combined interventions. Most relevant studies included CEA for selected intervention combinations based on a modelling of costs and effectiveness, including: (i) a decision-tree model evaluating the cost-effectiveness of malaria control measures in Africa [4]; (ii) an evaluation of the cost-effectiveness analysis of malaria control strategies in the context of the Millennium Development Goals (MDGs) using the World Health Organization (WHO)-CHOICE model [5]; and, (iii) a cost-effectiveness analysis of malaria control interventions in Kenya, which used the OpenMalaria stochastic simulation modelling platform [6].

This study presents a CEA of the various combinations of preventive and curative interventions, referred to as packages, implemented in Senegal during the period 2013–2014.

The objectives of this analysis are to:

- Estimate costs, effectiveness and cost effectiveness for different packages of malaria control interventions in Senegal using routine national programme and health information system data, as opposed to modelling;
- Compare costs and effectiveness across packages implemented in malaria control areas to identify potential efficiency gains and to draw lessons as malaria epidemiology continues to evolve in Senegal;
- Compare the cost-effectiveness ratios (CER), measured as the cost per disability adjusted life-year (DALY) averted, to the WHO GDP threshold.

The analysis results demonstrate a methodological approach (use of routine country data) and identify recommendations to support decision-making around value for money for the current distribution of packages. The question health policy makers face is how to best use the resources they have and, more importantly, how to demonstrate this good use of resources to other national or international stakeholders, so that they can attract more domestic and external resources to maintain or expand the current packages.

This analysis used the available data at the time of the study. As the Health Management Information System (HMIS) continues to improve in Senegal, this study could serve as a baseline of the costs and effectiveness of malaria control strategies to inform scale-up or changes in the current packages.

### Malaria control in Senegal

Malaria is endemic in most of Senegal, and nearly all 15 million inhabitants are at risk of the disease. The country can be divided into two epidemiological zones: the tropical zone with year-round transmission and the Sahelian zone with high transmission towards the end of and immediately after the rainy season. *Plasmodium falciparum* is the major malaria parasite species, accounting for more than 90% of all infections. Reported incidence of confirmed malaria per thousand persons slightly increased from 14 in 2009 to 19 in 2014. Overall incidence ranges from fewer than 5 per 1000 in some northern (Sahelian) districts to more than 200 per 1000 in some southeastern (tropical) districts [7].

The NMCP, together with its partners, implements a variety of malaria control interventions throughout the country, primarily with the financial support of the US Government through the President’s Malaria Initiative (PMI) and the Global Fund. During the study period (2013–2014), the NMCP was continuously implementing the following interventions:

1. Long-lasting insecticide-treated nets (LLINs): the national strategy includes a goal of universal coverage of LLINs through mass campaigns and routine distribution during antenatal clinics and immunization campaigns;
2. Indoor residual spraying (IRS) is conducted in selected districts based on incidence and entomological data. During the study period, 4 PMI target districts (Koungheul, Malem Hoddar, Velingara, Koumpentoum) received IRS in hotspots where the incidence was greater than 15 malaria cases per 1000;
3. Intermittent preventive treatment of malaria in pregnancy (IPTp) is conducted at all health facilities offering antenatal care (ANC) nationwide. According to the national strategy, all pregnant women should receive at least three doses of sulfadoxine–pyrimethamine (SP) starting in the second trimester, with at least 1 month between doses;
4. Seasonal malaria chemoprevention (SMC) involves an intermittent treatment of children aged 3–120 months with a combination of SP and amodiaquine (AQ). SMC is currently implemented in 16 high transmission districts meeting WHO SMC criteria.^1^
5. Case management: all patients receive rapid diagnostic tests (RDTs) and artemisinin combination therapy (ACT) free of charge at all levels of the health system. Testing with microscopy is available at health centers and hospital laboratories;
6. *Prise en charge à domicile* (PECADOM): home-based management of malaria is implemented at the community level, where community health workers (*Dispensateur de soins à domicile*-DSDOM) confirm malaria cases with RDTs, provide treatment with ACTs, and refer complicated cases to health facilities. DSDOMs also identify and treat diarrhoea and pneumonia in children under 5 years as part of integrated case management;
7. Focal test and treat (FTAT) is a form of reactive case investigation (RCI) carried out in one northern district (Richard Toll).

**Fig. 1:**
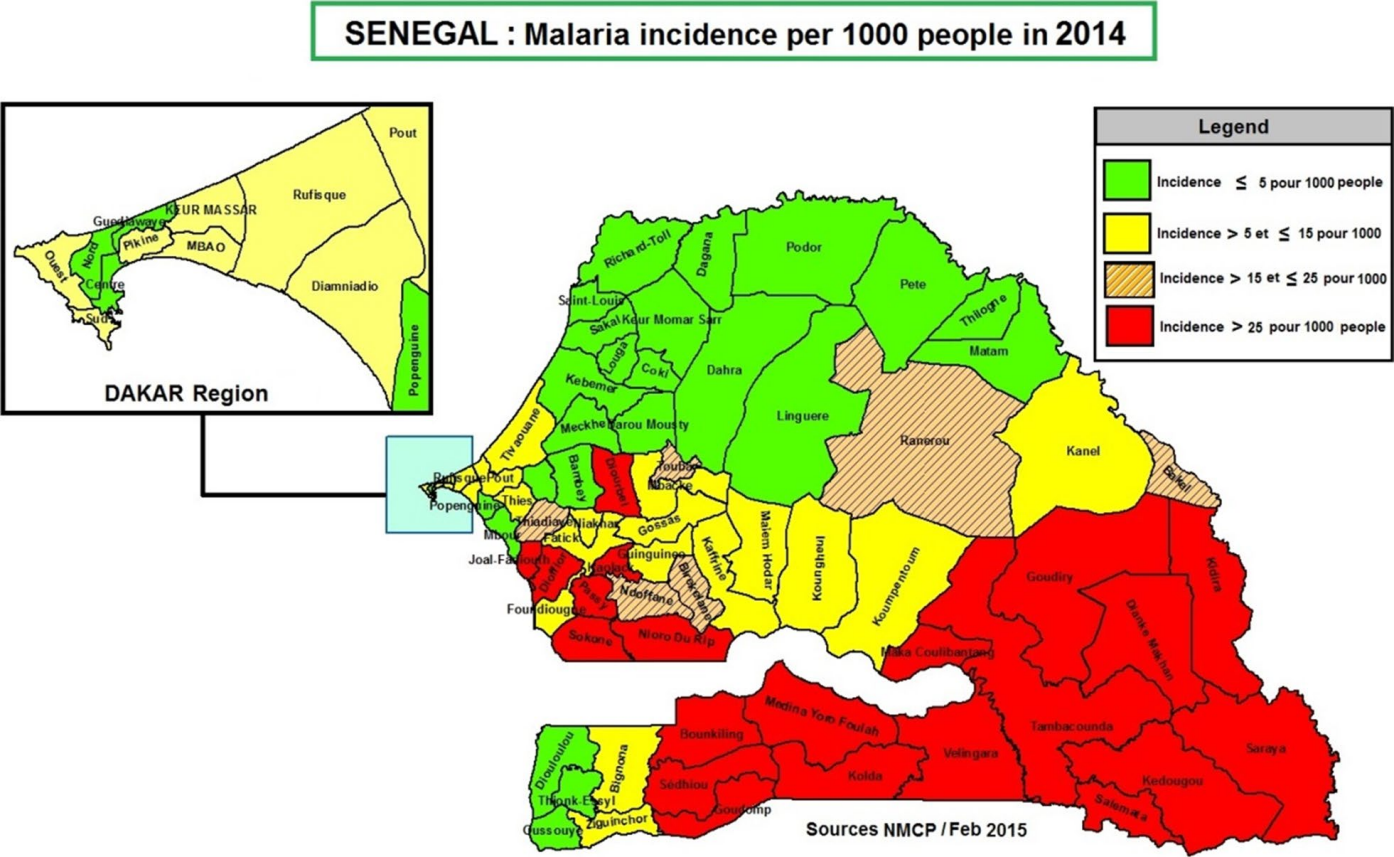
2014 map of malaria incidence

The NMCP strategy calls for deploying the described interventions in combinations/packages. Packages are implemented at the district level^2^ based on incidence level. Packages go from the basic scaling-up for impact (SUFI-only) to combinations of SUFI plus one or two other interventions. The SUFI package includes LLINs, IPTp, case management, and PECADOM, and is implemented nationwide. Such a package is intended to scale-up current proven interventions (such as LLINs) and is essential to controlling the epidemic in areas with high or moderate transmission but is also needed in low transmission settings to preserve gains. The other packages at the time of the study can be grouped into: (i) packages implemented in control districts (districts with moderate to high incidence) including SUFI + SMC (14 districts), SUFI + IRS (2 districts), and SUFI + IRS + SMC (2 districts); and, (ii) SUFI + FTAT package implemented in one elimination district (low incidence district, fewer than 5 cases per 1000 population). The labelling of control versus elimination districts was obtained from the NMCP. Figure 1 shows the malaria incidence map of the country at the time of the study.

The NMCP also coordinates other activities as part of its malaria control strategic plan: surveillance and monitoring through the continuous Demographic and Health Survey (cDHS) and through epidemic surveillance sites; entomological and insecticide resistance monitoring; social and behavior change communication campaigns; and operational research to provide data for decision making. During the study period the NMCP was piloting PECADOMPlus in 3 very high transmission districts (more than 200 cases per 1000 population). PECADOMPlus uses the same platform as PECADOM but involves active case management by DSDOMs, who visit every household in their communities once a week during the high transmission season (July to December) to test, treat or refer cases. In the north, MACEPA (Malaria Control and Elimination Partnership in Africa) also piloted mass test and treat (MTAT) in 3 districts with low transmission. In this intervention, all individuals within a specified target area were offered testing and treatment at the start of the transmission season. For more information on Senegal’s malaria control strategy, refer to the NMCP 2016–2020 strategic plan [8].

## Methods

### Design

Data on the costs and malaria burden were used to estimate cost effectiveness (cost per DALY averted) for the different packages. The study period was 2013–2014. The analysis was conducted from the perspective of the health system; there were not enough resources and time to capture costs incurred by patients and the analysis is mainly intended to inform NMCP decision making. Only financial costs were considered; for example, the opportunity cost of free labor from the unpaid community health workers is not accounted for. An assumption is made that, all else equal, the continuous implementation of a package over time should be the main contributor to a decrease in malaria burden. It is also assumed that the coverage of a package is the main determinant of its cost over time.

### Data collection

Data collection took place in Senegal during January and February 2016. Cost data was obtained from NMCP and implementing partners for each intervention. The data included all financial implementation costs (personnel, management, equipment, supplies, drugs, etc.) that could be directly linked to the interventions or allocated across several interventions. International headquarters offices costs were excluded as it was not possible to obtain these costs from all implementing partners. In the case of IRS, those costs were obtained, and only represented around 5% of the total intervention cost; hence we do not believe that their exclusion is seriously affecting the cost. Also not captured were the implementing partners’ local headquarters overhead costs (including rent, utilities, maintenance) when shared with many other health programmes. For example, the IntraHealth local office was supporting LLINs mass campaigns for the study period but also supports many other health interventions in the country. Although this could underestimate costs, it is not believed to have a big effect on the final cost measures as most of the interventions considered (except IRS and LLIN campaigns) were directly implemented by the NMCP with financial support from partners.

The NMCP also provided data on the number of malaria cases and number of malaria-related deaths for 2013 and 2014 from its routine malaria information system. Data was obtained on other variables that could affect malaria burden (i.e., household possession of nets and the distribution of health providers) from the country HMIS and the cDHS surveys. Estimates of health district monthly rainfalls (mm) were based on the Tropical Rainfall Measuring Mission (TRMM) 34B3 Monthly Rainfall Product (Version 7)^3^ [9]. Coverage or output data for each intervention was obtained from the NMCP routine malaria information system or directly from implementing partners. Depending on curative or preventive interventions, the data included the number of individuals protected by intervention or the volume of services provided by the intervention. All data was disaggregated by district. The output measures for each intervention and their data sources are in Table 1.

**Table 1:** Output measures and data sources for each intervention

Intervention	Output measures	Data sources
LLINs	Number of nets distributed through mass campaigns and routine distribution	cDHS, monitoring and evaluation (M&E) reports/routine HMIS data
IPTp	Number of pregnant women who received at least two doses	Annual malaria bulletin/routine HMIS data
RDTs/ACTs	Number of cases tested/number of cases confirmed and treated (health facility)	Annual malaria bulletin/routine HMIS data
PECADOM	Number of cases tested/number of cases confirmed and treated (community)	Annual malaria bulletin/routine HMIS data
SMC	Number of children protected who received the required number of doses	M&E reports/routine HMIS data
IRS	Number of structures sprayed/number of individuals protected	PMI Africa Indoor residual spraying (AIRS) project M&E data and annual malaria bulletin
FTAT	Number of cases tested/number of cases confirmed and treated	Annual malaria bulletin/PATH-Malaria Control and Elimination Partnership in Africa (MACEPA) M&E data

### Analysis

#### Package costs estimation

Five cost categories were used to estimate total and unit costs for each intervention: personnel, training, distribution,^4^ consumables,^5^ and monitoring and evaluation (M&E). Total costs for each intervention were obtained using a mix of top–down and bottom–up approaches [10]. Costs were converted from local currency to US$ using the 2014 average exchange rate.^6^ For LLIN mass campaigns, a standardized annual cost was estimated, given that mass distribution was occurring on average every 3 years for a group of geographically close districts. The cost of a package deployed in a particular area was obtained by using the cumulative costs of the interventions included in that package. Using output data on the coverage of preventive interventions, a unit cost per person protected was calculated. A unit cost per capita for each package was also estimated using the entire population of the areas where interventions are implemented. Unit cost data were then compared to identify potential efficiency gains.

#### Package effectiveness estimation

The analysis measured effectiveness of the packages implemented at the district level by the number of DALYs averted per 1000 population over the study period [11]. Because of the mix of preventive and curative interventions in each package, the DALYs averted were used to obtain a common measure of effectiveness. DALYs are the sum of the present value of future years of lifetime lost through premature mortality (years of life lost, or YLLs), and the present value of years of future life lived with any mental or physical disability caused by a disease or an injury (years of ‘healthy’ life lost, or YLDs). Following formulas described by Fox-Rushby and Hanson, DALYs represent a weighted combination of incidence and mortality. They therefore are not desirable, and the goal is to reduce or avert them [12].

For incidence estimation, a method combining routine HMIS/programme data and cDHS data, proposed by Cibulskis et al. [13], was used. The 2011 World Malaria Report approach [14], which employs the number of cases and the case fatality rate for malaria, was used to estimate mortality.

To calculate the number of DALYs, Senegal age-specific life expectancy was obtained from the WHO country life tables [15]. The Global Burden of Disease 2010 [16] disability weights were used and, following that study’s recommendations, social preferences (discounting and age weighting) were not applied. Following Gunda et al. [17], the average duration of disease used was 7 days (0.02 years).

### Package cost-effectiveness

The cumulative costs of a package in its area of implementation were divided by the number of DALYs averted between 2013 and 2014 to define the package’s cost effectiveness. The results were compared with the threshold that WHO’s Choosing Interventions that are cost-effective (WHO-CHOICE) project recommended and which was used by Mueller et al. [18, 19]. Those standards regard interventions with a cost per DALY averted less than the country’s per capita gross domestic product (GDP) as very cost-effective and those with a cost per DALY less than 3 times GDP per capita as cost-effective. Senegal’s GDP per capita in 2014 was US$1067.2 [20]. Results were also compared across packages, as appropriate; to assess the relative cost effectiveness among them.

### Sensitivity analysis

A univariate sensitivity analysis was done for effectiveness (DALYs averted) and costs. As mentioned above, for incidence estimation a method based on routine surveillance data proposed by Cibulskis et al. [13] was used. The method produced an upper and lower limit for incidence by varying the parameters used in the calculation. The case fatality rate was then applied to these two values to get the corresponding upper and lower limit values for mortality. DALYs were calculated with these values to get a range for effectiveness.

Because most of the intervention costs are recurring annual costs and were obtained from primary data collected from implementing partners, minimal uncertainty in their true value was anticipated. LLINs mass campaign is the exception as campaigns happened on average every 3 years, hence we estimated an annualized cost for this analysis. For sensitivity analysis a 2 years gap between campaigns is considered and a corresponding annual cost is estimated. We chose to focus on LLIN for cost sensitivity analysis because LLIN campaigns aim for universal coverage and are part of all packages, they account for an important part of the total cost of malaria control in Senegal (around 50%) and can significantly affect cost effectiveness.

### Determinants of package effectiveness (incidence and mortality)

The package of interventions considered should logically lead to a decrease in malaria burden (incidence and mortality) to the extent that coverage is effective. However, there could be other factors that affect malaria burden. Separate negative binomial regressions were used, with population as an exposure variable, to assess the effect of the type of package, the density of health providers, the coverage of IPTp, the coverage of LLINs, and rainfall level on the change in incidence and mortality over the study period. The negative binomial was used instead of the ordinary least squares (OLS) regression because the number of malaria cases/deaths cannot be negative and therefore normal distribution does not apply. The analysis focused on the packages deployed in the control districts (and excludes SUFI + FTAT) as most of the country is currently in a control stage. IPTp and LLIN coverage were chosen as co-variates because they are the only two preventive interventions implemented nationwide. The LLIN coverage represents the proportion of households having 1 LLIN for every 2 people. IPTp coverage represents the percentage of pregnant women who received a second dose of SP. Most interventions are deployed totally or partially through the current health system and will be affected by the density of health providers. The level of rainfall affects the mosquito life cycle, hence the burden of malaria.

To illustrate the effect of the control packages in the districts where they were implemented compared to the situation of SUFI only, predictions from the negative binomial regressions were used to estimate the incremental DALYs averted over the period 2013–2014 for packages including SMC or IRS. In the absence of effectiveness data before SMC and IRS were added to SUFI, this was an attempt to portray the baseline situation in those districts and compare it with the current situation.

## Results

### Coverage and costs

Among the interventions assessed, LLINs had the lowest coverage (65.9%) within the target population (Table 2). For IPTp, 66.3% of pregnant women who attended ANC received at least two doses of SP. IRS (96.3%) and SMC (97.2%) had the highest coverage levels of the 4 prevention interventions. Note that coverage, defined as relative to a pre-set target, was not applicable for curative interventions as treatment is provided to all sick individuals seeking care.

**Table 2:** Intervention coverage and unit costs

Intervention type	Coverage: % of individual protected compared to target	Unit costs per beneficiary (USD)
Prevention		
IRS	96.3	3.57
SMC	97.2	2.38
LLIN	65.9	1.64
IPTp	66.3	0.56
Treatment		
PECADOM	NA	5.90
FTAT	NA	6.46
Case management	NA	1.43

Curative interventions targets are not pre-set. Treatment is provided to all sick individuals seeking for care. LLIN coverage is for possession from mass campaigns and routine distribution. IPTp coverage is for the second dose. Case management represents facility-based case management as opposed to community-based (PECADOM)

**Fig. 2:**
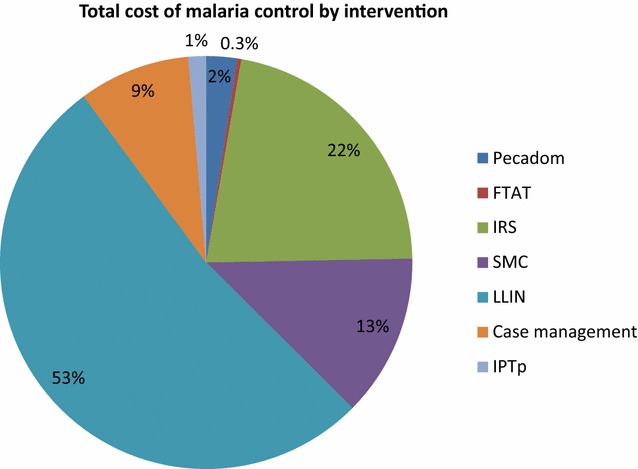
Distribution of total costs by intervention

In terms of total costs of interventions (Fig. 2), LLIN distribution (implemented nationwide, through both mass campaigns and routine distribution) was the most costly, accounting for 53% of total costs. IRS (implemented in 4 districts) had the second highest costs (22% of total costs), followed by SMC (implemented in 16 districts) at 13% of total costs. When accounting for coverage (Table 2), LLINs ($1.64) and IPTp ($0.56) had the lowest unit cost per beneficiary among preventive interventions. At $3.57 per person protected, IRS had the highest unit cost among preventive interventions, followed by SMC ($2.38). FTAT had the highest unit cost per beneficiary among treatment interventions ($6.46) followed by PECADOM ($5.90) and case management of uncomplicated malaria at health facilities ($1.43).

In terms of packages (Table 3), the ones with IRS have the highest per capita cost: the SUFI + IRS + SMC package cost $4.51 per person, followed by SUFI + IRS ($4.13 per person). The SUFI + SMC package cost $1.44 per person and SUFI + FTAT had a per capita cost of $0.68. SUFI-only has the lowest unit cost per capita ($0.51). The combined yearly unit cost at the country level is $0.82 per capita.

**Table 3:** Costs by malaria intervention package over the period of 2013–2014

Packages	Number of districts	Cumulative population of coverage areas	Total cost (thousands USD)	Unit cost per capita (USD)
SUFI + IRS + SMC	2	441,530	1,990,925	4.51
SUFI + IRS	2	289,594	1,194,947	4.13
SUFI + SMC	14	1,579,067	2,270,379	1.44
SUFI + FTAT	1	166,428	112,990	0.68
SUFI only	57	11,600,000	5,941,093	0.51
Total	76	14,076,619	11,510,334	0.82

### Disease burden

There was a decrease in malaria burden from 2013 to 2014 for all indicators (Table 4): incidence declined 33% on average, mortality declined 50% on average, and DALYs fell 46% on average per 1000 population. The SUFI + IRS + SMC package had the largest decrease in incidence rate (− 52%), mortality rate (− 90%) and DALY rate (− 88%).

**Table 4:** Malaria burden of disease changes over the period of 2013–2014 by package

Package	Number of districts	Average 2013 incidence rate^a^	Change in average incidence rate (%)	Average 2013 mortality rate^a^	Change in average mortality rate (%)	Average 2013 DALY rate^a^	Change in average DALY rate (%)
SUFI only	57	36.7	− 33	0.07	− 43	4.48	− 33
SUFI + IRS	2	29.2	− 38	0.11	− 82	6.84	− 78
SUFI + SMC	14	324.8	− 32	0.43	− 51	27.23	− 51
SUFI + IRS + SMC	2	79.8	− 52	0.29	− 90	17.68	− 88
SUFI + FTAT	1	3.08	− 45	0.01	− 42	1.17	− 39

^a^Incidence, mortality, and DALY rates are respectively in number per 1000 population

Table 5 shows the incidence rate ratios (IRR) for the negative binomial regression. The density of public health providers is not significantly related to incidence or mortality. The level of rainfall significantly (p < 0.05) affects both the number of malaria cases and the number of malaria deaths over the period: both measures increase as rainfall level increases. LLIN coverage (in terms of possession) is significantly (p < 0.05) and negatively associated with the number of malaria cases but has no significant effect on the number of malaria deaths. IPTp coverage is significantly (p < 0.05) and positively associated with the number of cases, with no effect on the number of deaths. This result could be due to additional efforts made to expand IPTp coverage in the areas with high malaria transmission or to other variables specific to those areas.

**Table 5:** Regression analysis of changes in malaria incidence and mortality over the period of 2013–2014

Variables	Change in incidence*	Change in mortality
Constant	3.93	6.9E−12
	(30.53)	(9.22E−11)
Public provider density	0.93	1.15
	(0.06)	(0.14)
LLIN coverage (possession)	0.46	1.90
	(0.17)**	(1.36)
IPTp coverage	1.24	0.84
	(0.11)**	(0.16)
Rainfall level	1.00	1.001
	(0.0003)***	(0.0007)**
Package categorical variable (change compared to SUFI only)		
SUFI + SMC	0.68	0.39
	(0.08)***	(0.08)***
SUFI + IRS	1.03	0.29
	(0.28)	(0.17)**
SUFI + IRS + SMC	0.49	0.12
	(0.14)**	(0.05)***
Log likelihood	− 1283.25	− 390.33
Pseudo R squared	0.1748	0.3128
Prob > chi^2^	0.000	0.000
Nbr. observations	152	152

* We reported the IRR (incident rate ratio) instead of traditional coefficients. Standard errors are reported in parentheses. ** and *** significance at respectively 5 and 1%

Regarding the packages’ effects compared with the SUFI-only, the packages implemented in control districts are associated with a further decrease in malaria incidence and mortality. Compared with SUFI-only, the SUFI + SMC (p < 0.01) and SUFI + IRS + SMC (p < 0.05) packages are associated with significant decreases in both incidence and mortality. Compared with SUFI-only, SUFI + IRS is associated with a significant decrease in mortality (p < 0.05) but no significant effect on incidence.

The simulation results for the incremental effect of adding IRS and/or SMC compared with SUFI-only, using predictions from the negative binomial regression, are shown in Table 6. Adding IRS to SUFI led to a 70% decrease in predicted DALYs, and adding SMC to SUFI was associated with a 60% decrease. When both SMC and IRS are added to SUFI, the DALYs are predicted to decrease by 85% compared to the SUFI-only situation.

**Table 6:** Predicted values of DALYs for observed package compared to SUFI only

Packages	2014 predicted DALYs under SUFI only scenario	2014 predicted DALYs under observed program	Incremental DALYs averted for the period 2013–2014	Percent decrease in DALYs (%)
SUFI + IRS	1580	485	1094	70
SUFI + SMC	39,959	15,967	23,992	60
SUFI + IRS + SMC	8160	995	6943	85

### Package cost-effectiveness

The cost per DALY averted shows wide variation across packages, ranging from $76 to $1591 (Table 7). With the sensitivity analysis, the cost per DALY averted can go as low as $61 and as high as $3237. Using WHO’s GDP threshold, it can be concluded that all packages are ‘very cost effective’ (the cost per DALY averted is less than the GDP per capita of $1067), except for the SUFI + FTAT package. However, the SUFI + FTAT package can still be considered cost effective because its cost per DALY averted is less than three times the GDP per capita, except when using the upper value on the sensitivity analysis.

**Table 7:** Cost effectiveness (cost per DALY averted) by malaria package

Packages	Cost per DALY averted	Sensitivity analysis	
		Lower value	Upper value
SUFI only	130	103	235
SUFI + IRS	582	456	836
SUFI + SMC	76	61	113
SUFI + IRS + SMC	272	217	376
SUFI + FTAT	1591	1119	3237

Comparing cost effectiveness across packages implemented in control districts, SUFI + SMC is the most cost-effective package with a cost per DALY averted of $76. Compared with SUFI + IRS, the package with added SMC (SUFI + IRS + SMC) had a lower cost per DALY averted ($272 versus $582). These results seem to suggest that adding SMC to IRS (when applicable) enhances its cost effectiveness.

The SUFI + FTAT package, implemented in elimination districts, has a cost per DALY averted of $1591, which is almost at the GDP threshold. There was no other package implemented in elimination districts, hence no comparison to identify the one giving more value for money in that situation. However, the findings about SUFI + FTAT provides some insights into the short-term costs and corresponding outcomes of malaria interventions targeting low transmission areas where there are few cases but where an important surveillance system is needed to avoid resurgence of the disease.

SUFI-only is a “very cost effective” package with a cost of $130 per DALY averted. However, this result hides variations in the group of 57 districts where this package was implemented. Not all districts with SUFI-only saw a decrease in DALYs. A rapid efficiency-scenario showed that if DALYs in districts with no decrease went down at the same rate as that observed in their peer-districts, the cost per DALY averted could further decrease to $109 (16% decrease from the current value). A further analysis of the SUFI-only group will be needed to better understand how some of the districts could benefit from some of the other current packages.

## Discussion

There are multiple interventions available for the fight against malaria. Most of them are effective at decreasing malaria burden [21–25]. However, no single (currently available) intervention will achieve malaria elimination. A package of interventions must be rolled out as appropriate along the spectrum of malaria transmission intensity [26]. Studies have found that implementing combinations of interventions seems to enhance the interventions’ individual effectiveness [27]. Given the goal of scale-up, comprehensive malaria control strategies are then more effective [28]. Building on recommendations from the roll back malaria (RBM) action plan [29], Senegal’s NMCP has defined packages using different malaria interventions and deployed them across the country. This study assessed the cost per DALY averted for the different packages that the NMCP in Senegal used during the period 2013–2014. The results suggest that the programme’s package targeting is effective, as the country’s malaria burden decreased during the study period, as evidenced by declines in incidence, mortality and DALYs at the country level of 33, 50 and 46%, respectively. Moreover, the study findings show that all packages used in Senegal are either very cost effective or cost effective according to the WHO thresholds. This model of scaling-up interventions through package deployment guided by the incidence level seems to work in Senegal and could be an example for other countries that are mostly in the malaria control stage. However, it should be noted that the WHO threshold of GDP per capita is not widely accepted as a reasonable measure of cost effectiveness and more analysis (for example, budget analysis) should be done before scaling up the packages.

The NMCP is currently planning to eliminate malaria in the north of the country, where malaria incidence is mostly low (fewer than 5 cases per 1000 people). FTAT is, therefore, implemented (for now in 1 district) in addition to SUFI to ensure that the few (and mostly imported) cases of malaria are identified and rapidly treated to avoid transmission. Findings from this study suggest that FTAT has the highest unit cost per beneficiary among all interventions ($6.46). The finding of malaria elimination requiring more resources (in terms of unit costs) in the short term compared to malaria control has already been assessed [30]. This study gives insights into the magnitude of costs and corresponding outcomes for Senegal in particular, and can help the NMCP plan and advocate for the required level of resources as the SUFI + FTAT package is expanded in more low-transmission districts. There is currently no optimal package of interventions for elimination. Interventions will have to be context-specific and tailored to particular countries, making it crucial for those countries to have customized cost and outcomes information.

Moreover, the fact that the initial costs of elimination could likely be higher than those of a control programme can pose a problem for resource allocation [31, 32]. This problem is more pronounced for countries, such as Senegal, with both elimination and control zones. Gonzalez-Silva et al. write that the first step in moving the malaria eradication agenda forward “will be to convince all involved parties that malaria control and elimination are not mutually exclusive.” [33].

As seen from the rapid efficiency-scenario results, the SUFI-only package can further benefit from potential efficiency gains if DALYs decreased in all districts (16% decrease in the cost per DALY averted compared with the current situation). The relatively low coverage of some SUFI interventions, particularly LLINs (average coverage 66%), possibly accounts for the fact that the SUFI package still has room for improvement. Indeed, the regression results suggest that LLIN coverage significantly affects both incidence and mortality, hence DALYs, so expanding its coverage could increase effectiveness. However, it is possible that some ‘programme/district management’ level variables that could not be accounted for in the regression may also have an important influence on effectiveness. Working towards increasing the coverage of LLINs and addressing those other issues related to programme/district management could possibly increase the cost-effectiveness of the current packages, particularly for SUFI-only.

Evidence is key when designing and implementing any health strategy, and it is no different for malaria. Without a system for collecting data on disease surveillance and intervention outcomes, it will be difficult to have a relevant country strategy. Strong surveillance systems are particularly important for the elimination phase as case identification needs to be rapid and efficient. The NMCP in Senegal has put in place over the years a system for collecting relevant malaria data throughout the country, and those data are the main enablers for studies such as this one, which try to align with the country context as much as possible. Moreover, an already strong information system in the control phase can mean less initial investment in information systems when transitioning to an elimination phase.

This study provides some of the needed cost and effectiveness data to guide policy decisions in malaria control in Senegal, but it has limitations. The study focused on a short period, 2013–2014, and the coverage of LLINs during this period was relatively low. The study did not include the start year of interventions/packages as not everything started at the same time and not all historical data were available.^7^ The objective was to provide the NMCP with a snapshot CEA of the current packages, assuming their continuous implementation in the short term. As such, this analysis can serve as a baseline and reference for future studies looking at the cost effectiveness of these packages as the epidemiological profile of the country and intervention/package coverage evolve.

For the cost estimations, due to data limitations this study did not account for costs that did not represent a direct investment into intervention implementation. This is a limitation as it can underestimate the total cost of interventions such as IPTp and case management, which are implemented through the existing health system (health facilities). Nevertheless, this study is still useful to the NMCP as the marginal cost of implementing these interventions is what programme officers need for budgeting and planning purposes.

## Conclusion

Senegal and other countries contemplating malaria programme scale-up to achieve elimination need high-quality data to plan, budget and monitor their national programmes. The data must account for the local programming context and be routinely available. With a good surveillance and information system, CEA of malaria interventions based on actual country data, as opposed to trials or modelling, can give a more accurate picture of relative performance in real implementation settings. This study uses such an approach to assess the cost per DALY averted for the different packages used by the NMCP in Senegal during the period 2013–2014.

Most important, the study findings can be used to increase the efficiency of the current programme and guide the efficient scale-up of the programme in the future. The evidence documented in this study suggests that targeting specified malaria profile zones with a package of interventions is effective, and most of the packages that Senegal currently uses are very cost effective. The SUFI + SMC package is very cost effective in areas with high malaria burden where it is currently implemented, and could possibly be expanded to other high transmission districts where applicable and consistent with WHO recommendations. Some districts currently receiving SUFI-only could be good candidates for SMC expansion. Following the presentation of this study's results, NMCP managers expressed their desire to expand SMC (if funding is available) given the evidence of not only effectiveness but cost-effectiveness. The relatively high unit cost per beneficiary for the SUFI + FTAT package implemented in elimination zones suggests that more resources will be needed as additional parts of the country enter the elimination phase.

The packages of interventions used in Senegal worked in terms of decreasing malaria burden but more research is needed to better understand the determinants of effectiveness of those packages beyond coverage. A better knowledge of those parameters will help enhance package outcomes, increase efficiency and realize potential savings, which Senegal could invest to achieve its malaria elimination goal.

## Abbreviations

ACT: artemisinin combination therapy
AQ: amodiaquine
cDHS: continuous Demographic and Health Survey
CEA: cost effectiveness analysis
DALY: disability adjusted life years
DSDOM: community health workers for malaria treatment
FTAT: focal test and treat
GDP: gross domestic product
IPTp: intermittent preventive treatment of malaria in pregnancy
IRS: indoor residual spraying
LLIN: long-lasting insecticide-treated nets
NMCP: National Malaria Control Programme
PECADOM: home-based management of malaria
PMI: President’s Malaria Initiative
RDT: rapid diagnostic tests
SMC: seasonal malaria chemoprevention
SP: sulfadoxine–pyrimethamine
SUFI: scaling-up for impact
WHO: World Health Organization

## Footnotes

1 At least 60% of cases occur within 4 months of the year, including at least 10% annual incidence among children.

2 The district is the smallest administrative subdivision in the Senegalese health system.

3 TRMM 3B43 data contain the best-estimate precipitation rate (in mm/h) and root-mean-square (RMS) precipitation-error estimates from TRMM and other data sources.

4 Distribution costs refer to all logistic costs related to distributing the intervention: transport, communication, material, equipment (depreciated if applicable), and other logistics costs. Interventions implemented through the health system do not have separate distribution costs (case management, IPTp).

5 Consumable costs refer to the cost of tests and drugs for case management, PECADOM, IPTp, RCI, SMC; cost of nets for LLIN distribution; cost of insecticides for IRS.

6 Average exchange rate US$493 from https://www.ofx.com/en-us/forex-news/historical-exchange-rates/.

7 From 2010 to 2012, Senegal’s health workers were on strike, using data retention as a means of protest. Normal routine data collection resumed in 2013.
